# Changes in the Sensitivity of MCF-7 and MCF-7/DX Breast Cancer Cells to Cytostatic in the Presence of Metformin

**DOI:** 10.3390/molecules29153531

**Published:** 2024-07-27

**Authors:** Justyna Płonka-Czerw, Luiza Żyrek, Małgorzata Latocha

**Affiliations:** Department of Cell Biology, Faculty of Pharmaceutical Sciences in Sosnowiec, Medical University of Silesia, Jedności 8, 41-200 Sosnowiec, Poland; l.pastor@wp.pl (L.Ż.); mlatocha@sum.edu.pl (M.L.)

**Keywords:** breast cancer cells, multidrug resistance (MDR), metformin, doxorubicin, cytostatics

## Abstract

Multidrug resistance is a serious problem in modern medicine and the reason for the failure of various therapies. A particularly important problem is the occurrence of multidrug resistance in cancer therapies which affects many cancer patients. Observations on the effect of metformin—a well-known hypoglycemic drug used in the treatment of type 2 diabetes—on cancer cells indicate the possibility of an interaction of this substance with drugs already used and, as a result, an increase in the sensitivity of cancer cells to cytostatics. The aim of this study was to evaluate the effect of metformin on the occurrence of multidrug resistance of breast cancer cells. The MCF-7-sensitive cell line and the MCF-7/DX cytostatic-resistant cell line were used for this study. WST-1 and LDH assays were used to evaluate the effects of metformin and doxorubicin on cell proliferation and viability. The effect of metformin on increasing the sensitivity of MCF-7 and MCF-7/DX cells to doxorubicin was evaluated in an MDR test. The participation of metformin in increasing the sensitivity of resistant cells to the effect of the cytostatic (doxorubicin) has been demonstrated.

## 1. Introduction

Multidrug resistance is one of the most important causes of failures of cancer therapy. This situation is associated with various pharmacological and cellular conditions. These include mutations and deregulations within proteins and enzymes involved in the activation of chemotherapeutic drugs in cells and increased metabolism of xenobiotics or induction of antiapoptotic gene expression. There is also increasing evidence that cancer stem cells (CSCs) are involved in the development of MDR [1]. The most frequently observed changes in cell sensitivity to drugs are connected with the transport of drugs through membranes and cytostatic removal rate from the cell by membrane transport proteins.

Most of the proteins involved in the transport of drugs from the cell to the intracellular space belong to the family of ABC proteins. This type of proteins contains the ATP-binding domain (ATP-binding cassette family). A transport protein associated with drug resistance, but not belonging to the ABC family, is also described. This protein is defined as LRP (lung cancer resistance-related protein) [2]. It is located within the nuclear membrane and its role is probably related to cytostatic removal from the cell nucleus to the cytosol. Resistance to therapy is often associated with the simultaneous overexpression of MRP and LRP or that of MRP, LRP and P-gp [3].

Metformin, a well-known hypoglycemic drug, is mainly used in the treatment of type 2 diabetes. It reduces hepatic glucose production and sensitizes peripheral tissues to insulin. It stimulates the AMPK enzyme (AMP-dependent kinase), which leads to changes in the sensitivity of cells to insulin increases, peripheral glucose uptake and its metabolism in cells. It decreases the absorption of glucose in the gastrointestinal tract, reduces lipogenesis and stimulates the process of β-oxidation. It increases the sensitivity of cells to the action of incretin hormones, i.e., glucose-dependent insulin-like peptide (GIP) and glucagon-like peptide 1 (GLP1) [4]. The beneficial effects of metformin in cancer are related to the inhibition of the mTOR signaling pathway (by activating AMPK). This leads to, e.g., the inhibition of cell proliferation, which means that it can have an impact on inhibiting the growth of cancer cells but also play an important role in the aging processes of organisms [4,5]. The suppression of the mTOR pathway also leads to the induction of autophagy, which is attributed a significant role in the process of extending life. Studies conducted in vitro on cancer cells indicate the possibility of interactions of metformin with drugs already used by sensitizing cells to cytostatics [6,7,8,9,10,11,12].

The aim of this study was to assess the effect of metformin on the occurrence of multidrug resistance. The effect of metformin of breast cancer cells originating from the metastatic focus were analyzed: MCF-7-line human-breast-adenocarcinoma wild-line cells, which are sensitive to cytostatics, and MCF-7/DX human-breast-adenocarcinoma subline cells resistant to cytostatics.

## 2. Results

**Figure 1:**
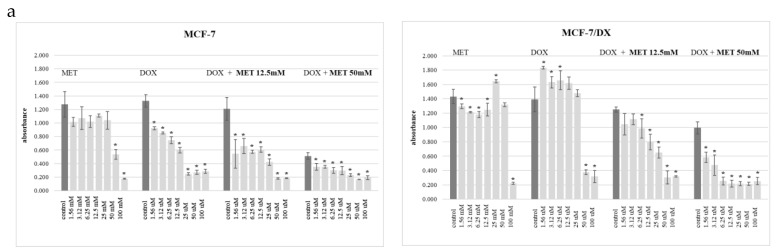

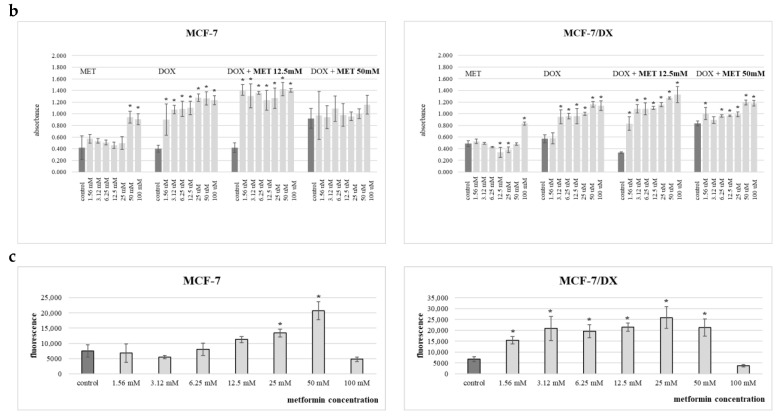
(**a**) The effect of different doses of metformin (1.56–100 mM), doxorubicin (1.56–100 µM) and mixture of doxorubicin (1.56–100 µM) with metformin (12.5 mM and 50 mM) on the metabolic activity of MCF-7 and MCF-7/DX cells (WST-1 assay). (**b**) The cytotoxicity effect of different doses of metformin (1.56–100 mM), doxorubicin (1.56–100 µM) and mixture of doxorubicin (1.56–100 µM) with metformin (12.5 mM and 50 mM) in the MCF-7 and MCF-7/DX in vitro cultures (LDH assay). (**c**) The effectiveness of different doses of metformin (1.56–100 mM) in overcoming the resistance of the MCF-7 and MCF-7/DX cells to doxorubicin (MDR assay). Statistical significance is set to *p* ≤ 0.05 (*).

The result of the WST test performed for cells of the MCF-7 line after 72 h of incubation showed the toxic effect of metformin at a concentration of 50 mM and 100 mM, and the toxic effect of doxorubicin depending on its concentration in the medium. An increase in the toxic effect of doxorubicin was also observed in the presence of metformin at both 12.5 mM and 50 mM. Under the same experimental conditions, the results obtained for the cells of the MCF-7/DX line indicate a sensitivity of the cells to metformin, with the highest decrease in absorbance at a concentration of 100 mM and doxorubicin (significant decrease in absorbance at 50 µM and 100 µM). Simultaneous exposure of MCF-7/DX to doxorubicin with the addition of metformin—both at a concentration of 12.5 mM and 50 mM—causes a decrease in the relative number of viable cells in the cultures (Figure 1a).

The results of the LDH tests confirm the dependencies obtained in the WST-1 tests and indicate the cytotoxic effect of the abovementioned drugs on cells in cultures (Figure 1b).

The effectiveness of metformin in overcoming the resistance of the tested cells to doxorubicin is shown in Figure 1c. The dye included in the MDR (Sigma-Aldrich, St. Louis, MO, USA) test quickly penetrates through the membrane into the cell. If not removed outside, it undergoes an enzymatic reaction and shows fluorescence. The increase in fluorescence of MCF-7/DX cell cultures exposed to metformin, in the concentration range from 1.56 mM to 50 mM, indicates an increase in the sensitivity of the tested cells to doxorubicin.

**Figure 2:**
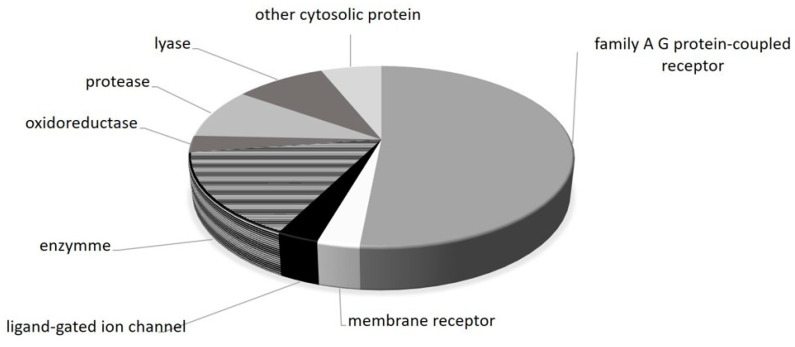
Diagram showing molecular targets for metformin determined from drug structure using the SwissTargetPrediction program.

Based on the molecular structure of metformin (Figure 2, Table 1), 33 potential targets of metformin were predicted using the SwissTargetPrediction online tool.

## 3. Discussion

One of the most important reasons for the failure of cancer therapy is the development of congenital or acquired drug resistance to chemotherapeutic agents. In the case of congenital resistance, the cancer cells do not respond to standard chemotherapeutic agents from the beginning of therapy, and in the case of acquired resistance, initially selected drugs are effective, but after a certain period of time, they develop resistance to drugs. Cells acquire cross-resistance to a wide range of drugs with different cellular targets and structures. Therefore, acquired resistance requires the use of ever-increasing concentrations of drugs, in high doses, waiting for a therapeutic effect, which, however, does not appear. The applied treatment becomes ineffective, harmful and destructive to the entire body.

The cellular mechanisms of MDR are defined by changes in the biochemistry of cancer cells consisting of reduced absorption of drugs, their rapid removal from the cell, changes in molecules that are drug targets and their rapid inactivation [2,3,13,14,15]. Classic transport-based mechanisms are associated with the activity of ABC family proteins containing the ATP-binding domain [2]. They are associated with rapid removal of the drug from the cells. Over the years, scientists around the world have been looking for solutions to the problem of drug resistance. One of the methods is the use of compounds that bind MDR transporters and block their effect. This results in sensitization of cells to the chemotherapeutic agents used. These compounds include pharmacological substances, monoclonal antibodies and immunotoxins [16,17,18]. Many studies have been related to the use of P-gp modulators blocking drug-binding sites within P-glycoprotein. The modulator is used together with a cytostatic drug. Due to the higher affinity or increased concentration achieved in the cell, the compound effectively competes with the drug for access to the transport protein. This leads to its saturation and significantly reduces the outflow of the drug from the cell.

The influence of the PI3K/AKT pathway on the development of multidrug resistance is also described in the literature. The signaling pathway of 3-phosphatidyloinositol and AKT protein kinase plays an important role in the development of MDR in various cancers, e.g., breast cancer, lung cancer, leukemia, ovarian cancer and melanoma [19,20,21]. The development of MDR may be related to the PI3K/AKT signaling pathway through activation of the NF-κB protein. NF-κB proteins in their active form function as regulatory transcription proteins. In the process of carcinogenesis, due to abnormal, constant activation of the NF-κB pathway, there is abnormal control of the expression of genes encoding proteins that play an important role in many processes at the cellular level, such as proliferation, differentiation, apoptosis and angiogenesis. Increased NF-κB transcriptional activity has a stimulating effect on the process of cancer cell proliferation by facilitating the transition of cells from the G1 phase to the S phase of the cell cycle as a result of transcription stimulation, among others, cyclin D1. Abnormal activation of the PI3K/AKT/NF-κB pathway also regulates P-gp expression, contributing to the development of MDR [19].

Metformin, a biguanide derivative, according to registration indications, is mainly used for the treatment of type 2 diabetes and for the prevention of various metabolic diseases [22,23]. Mechanism of action of this drug is primarily based on reducing the absorption of sugars in the intestines, inhibiting gluconeogenesis and glycogenolysis in the liver and increasing the sensitivity of cells to insulin (skeletal muscle, heart, liver and adipose tissue) without affecting its production. Molecularly, metformin inhibits respiration in the mitochondria at the level I complex of the respiratory chain [4,24]. The result is a shift in the energy balance of cells that increases AMP kinase activity. Many studies confirm that metformin is a substrate for cationic transporters such as OCT1 (SLC22A1), OCT2 (SLC22A2), OCT3 (SLC22A3), MATE1 (SLC47A1), MATE2 (SLC47A2), PMAT (SLC29A4) and OCTN1 (SLC22A4) [25].

In addition to the hypoglycemic effect, more and more attention is being paid to the beneficial effects of metformin in cancer therapy. The effect of metformin is largely attributed to its ability to modulate the course of the cell cycle, apoptosis, autophagy and inflammatory processes. Attention is also drawn to the possibility of acting on oxidative stress and epigenetic regulation. At the molecular level, the anticancer effect of metformin may be due to the activation of AMP kinase, which inhibits the mammalian target of rapamycin (mTOR). The protective effect independent of AMPK, in turn, is the effect on inflammatory pathways, insulin-like growth factor-1 or leptin. Attention is also drawn to the inhibition of the growth and proliferation of cancer stem cells [4,5,8,26,27,28]. There have also been reports of a beneficial effect of metformin in combination with anticancer drugs, especially in relation to cells resistant to cytostatics [29,30,31,32,33,34].

In the presented study, the effect of metformin on the occurrence of multidrug resistance of breast cancer cells MCF-7 and MCF-7/DX (a line resistant to cytostatics, including doxorubicin) was evaluated. Cell viability in cultures was estimated on the basis of the results of the WST-1 test for the presence of live, metabolically active cells (Figure 1a) and the LDH test for the detection of dead cells with damaged membranes in cell cultures, which release the cytosolic enzyme lactate dehydrogenase into the medium (Figure 1b). The toxic effect of metformin alone on MCF-7 cells was demonstrated only at concentrations above 50 mM and the toxic effect of doxorubicin dependent on its concentration in the medium. Enhancement of the cell toxic effect of doxorubicin was obtained in the presence of metformin (both 12.5 mM and 50 mM). The results of WST-1 obtained of MCF-7/DX cells under the same conditions as for MCF-7 cells show the following: lower sensitivity of MCF-7/DX cells to metformin (the highest decrease in absorbance at a concentration of 100 mM) and doxorubicin (visible decrease metabolic activity of cells only at the concentration of 50 and 100 µM) and a decrease in absorbance after the use of a mixture of doxorubicin and metformin (both after using a 12.5 mM and 50 mM solution, which itself has a slight effect on the viability of MCF-7/DX). However, when dosing metformin in vitro, it should be borne in mind that supraphysiological doses may produce off-target effects that do not reflect in vivo events. Concentrations of 0.1 mM to 100 mM are typically used in vitro studies of metformin effects; for comparison, in vivo concentrations of metformin in human serum at a mean oral daily dose of 1500 mg are 2–38 μM (steady state ranges are 15.5μM) [35,36,37]. In this study, the effect of metformin on increasing the sensitivity of MCF-7 and MCF-7/DX cells to doxorubicin was evaluated on the basis of the results of the MDR test (Sigma-Aldrich) which is recommended as screening tests for MDR pump inhibitors or for identifying cell lines with high MDR activity (Figure 1c). The fluorescence values obtained in the study for cells of the MCF-7 and MCF-7/DX lines exposed to metformin indicate an increased accumulation of the dye in the cells, especially visible in the case of the MCF-7/DX line. On the basis of the structure of metformin, the most important molecular targets for this molecule were analyzed (using the SwissTargetPrediction program) (Figure 2 and Table 1).

The results shown in Table 1 and Figure 2 do not indicate binding to ABC transport proteins. The main target here are carbonic anhydrases, oxidoreductases, nitric oxide synthases and family A G protein-coupled receptors. However, there have been reports of the possibility of affecting the synthesis of MDR-1 and BCRP proteins (through AMPK-independent mechanisms). It has been proven that in the case of MCF-7 cells, metformin can reduce the amount of proteins in MDR-1, BCRP, HIF1α, p53S392phos (compared to total p53) and S6KS411phos (compared to total S6K), and even the number of Erα receptors [35]. Researchers in Brazil have shown that the effect of metformin on MCF-7 resistance is related to oxidative stress. Microarray analyses showed that doxorubicin-resistant cells alter the expression of genes involved in oxidative stress (OS) and the TGF-β pathway. Metformin increases sensitivity to DOX-induced OS; it lowers the concentration of nitric oxide, nuclear NF-kB and Nrf2 and increases nuclear p53. An analysis of the IFN-α signaling pathway has shown that metformin may increase sensitivity to apoptosis [38].

## 4. Materials and Methods

### 4.1. Cell Cultures

This research was carried out on cultures of breast cancer cells from a metastatic focus (pleural effusion), which have been bought from the ATCC. Acquired lines are MCF-7 line human-breast-adenocarcinoma cells sensitive to cytostatics and MCF-7/DX human-breast-adenocarcinoma subline cells resistant to cytostatics. The loss of sensitivity to doxorubicin (Sigma-Aldrich) of MCF-7/DX cells was confirmed by the Multidrug Resistance Assay Kit (Sigma-Aldrich).

### 4.2. Evaluation of the Effect of Metformin on the Sensitivity of Cells to Doxorubicin

The cells were exposed to doxorubicin (Sigma-Aldrich) at a concentration of 1.56–100 µM and metformin (Sigma-Aldrich) at a concentration of 1.56–100 mM and a mixture of doxorubicin at a concentration of 1.56–100 µM with metformin at a concentration of 12.5 mM or 50 mM. After 72 h of incubation (37 °C, 5% CO_2_), cell viability in cultures was evaluated, and a MDR transporter activity test was performed.

### 4.3. Cell Viability

After 72 h of incubation with drugs, the WST-1 (Roche Diagnostics, Mannheim, Germany) test for cell viability and the LDH (Roche Diagnostics) test for metformin and doxorubicin cytotoxicity were performed.

#### 4.3.1. WST1 Test (Roche Diagnostics)

A colorimetric assay was used to determine the relative number of viable cells in cultures. WST-1 (tetrazolium salt) is converted by viable, metabolically active cells to soluble formazan. The amount of formazan formed directly correlates with the number of metabolically active cells in the culture. Absorbance was measured at λ = 450 nm and λ = 600 nm as reference wavelength. A UVM340 microplate reader Asys-Hitech GmbH UVM340 (Eugendorf, Austria) was used for absorbance measurements.

#### 4.3.2. LDH Test (Roche Diagnostics)

A colorimetric relative dead-cell assay based on the measurement of lactate dehydrogenase (LDH) activity released from the cytosol of damaged cells into the culture medium. Measurements of absorbance at λ = 450 nm and λ = 600 nm were used as the reference wavelength. A UVM340 microplate reader Asys-Hitech GmbH UVM340 (Eugendorf, Austria) was used for absorbance measurements.

### 4.4. MDR Test (Sigma-Aldrich)

The kit uses a hydrophobic fluorescent dye molecule to assess MDR activity in cells. The dye quickly penetrates cell membranes, which increases the fluorescence intensity (λex = 490/λem = 525 nm). In drug-resistant cells with MDR transporters, the dye is quickly excreted outside, which reduces the fluorescence intensity. The test is intended for the screening of MDR pump inhibitors or for the identification of cell lines with high MDR activity. Fluorescence intensity was measured at λex = 490/λem = 525 nm using a Triad LT Multimode Detector (Dynex Technologies, Chantilly, VA, USA).

### 4.5. Prediction of Potential Molecular Targets of Metformin

The possible interactions of metformin with various molecular targets were simulated using the SwissTargetPrediction program [39]. SwissTargetPrediction is a program for predicting macromolecular targets (human and animal proteins) of small molecules. It allows one to predict the molecular mechanisms of action of such a molecule on cells. SwissTargetPrediction is based on the so-called “principle of similarity”, which generally states that two similar molecules have similar properties. SwissTargetPrediction compares particles to 370,000 other known particles.

### 4.6. Statistical Analysis

Data distribution for each of trials met the requirements of normal distribution (assessed with Wilk–Shapiro test), which gave the basis for a statistical analysis conducted with use of parametric tests. The control group was compared with seven tested groups using Dunnett’s test, after prior confirmation (with an F-test in a variance analysis) of statistically significant differences in the analyzed means. Assumptions of variance homogeneity were checked with use of Levene’s test and Brown–Forsythe’s test. The data were presented as charts with the arithmetic mean for each sample and the corresponding standard deviations. All the tests were performed with a significance level of α = 0.05, using Statistica version 13.3 software.

## 5. Conclusions

The results obtained in this study indicate that metformin in combination with doxorubicin increases the sensitivity of cancer cells to cytostatics. It has a beneficial effect on eliminating the phenomenon of drug resistance of breast cancer cells. The effect is already visible at concentrations above 12.5 mM.

Based on the results obtained in this work and the descriptions of the effects of similar studies appearing in publications, it can be concluded that metformin is a promising tool in preventing and combating chemoresistance in cancer patients.

## Figures and Tables

**Table 1:** Molecular targets for metformin determined from drug structure using the SwissTargetPrediction program.

Query Molecule	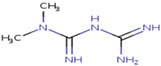	
Target	Target Class	Common Name
Adrenergic receptor alpha-2	Family A G protein-coupled receptor	ADRA2C
Alpha-1a adrenergic receptor	Family A G protein-coupled receptor	ADRA1A
Alpha-1b adrenergic receptor	Family A G protein-coupled receptor	ADRA1B
Alpha-1d adrenergic receptor	Family A G protein-coupled receptor	ADRA1D
Alpha-2a adrenergic receptor	Family A G protein-coupled receptor	ADRA2A
Alpha-2b adrenergic receptor	Family A G protein-coupled receptor	ADRA2B
Amine oxidase, copper containing	Enzyme	AOC3
Carbonic anhydrase I	Lyase	CA1
Carbonic anhydrase II	Lyase	CA2
Carbonic anhydrase IX	Lyase	CA9
Dihydrofolate reductase	Oxidoreductase	DHFR
Histamine H3 receptor	Family A G protein-coupled receptor	HRH3
Histamine H4 receptor	Family A G protein-coupled receptor	HRH4
Indoleamine 2,3-dioxygenase	Enzyme	IDO1
Integrin alpha-V/beta-3	Membrane receptor	ITGAV ITGB3
Nischarin	Other cytosolic protein	NISCH
Nitric oxide synthase, inducible	Enzyme	NOS2
Nitric oxide synthase, brain	Enzyme	NOS1
Nitric oxide synthase, endothelial	Enzyme	NOS3
S-100 protein beta chain	Other cytosolic protein	S100B
Serotonin 1a (5-HT1a) receptor	Family A G protein-coupled receptor	HTR1A
Serotonin 1b (5-HT1b) receptor	Family A G protein-coupled receptor	HTR1B
Serotonin 1d (5-HT1d) receptor	Family A G protein-coupled receptor	HTR1D
Serotonin 2a (5-HT2a) receptor	Family A G protein-coupled receptor	HTR2A
Serotonin 2b (5-HT2b) receptor	Family A G protein-coupled receptor	HTR2B
Serotonin 2c (5-HT2c) receptor	Family A G protein-coupled receptor	HTR2C
Serotonin 3a (5-HT3a) receptor	Ligand-gated ion channel	HTR3A
Serotonin 5a (5-HT5a) receptor	Family A G protein-coupled receptor	HTR5A
Serotonin 6 (5-HT6) receptor	Family A G protein-coupled receptor	HTR6
Serotonin 7 (5-HT7) receptor	Family A G protein-coupled receptor	HTR7
Thrombin	Protease	F2
Thrombin and coagulation factor X	Protease	F10
Urokinase-type plasminogen activator	Protease	PLAU

ADRA2C—adrenergic receptor alpha-2, ADRA1A—alpha-1a adrenergic receptor, ADRA1B—alpha-1b adrenergic receptor, ADRA1D—alpha-1d adrenergic receptor, ADRA2A—alpha-2a adrenergic receptor, ADRA2B—alpha-2b adrenergic receptor, AOC3—amine oxidase, copper containing, CA1—carbonic anhydrase I, CA2—carbonic anhydrase II, CA9—carbonic anhydrase IX, DHFR—dihydrofolate reductase, HRH3—histamine H3 receptor, HRH4—histamine H4 receptor, IDO1—indoleamine 2,3-dioxygenase, NISCH—nischarin, NOS2—nitric oxide synthase, inducible, NOS1—nitric oxide synthase, brain, NOS3—nitric oxide synthase, endothelial, S100B—S-100 protein beta chain, HTR1A—serotonin 1a (5-HT1a) receptor, HTR1B—serotonin 1b (5-HT1b) receptor, HTR1D—serotonin 1d (5-HT1d) receptor, HTR2A—serotonin 2a (5-HT2a) receptor, HTR2B—serotonin 2b (5-HT2b) receptor, HTR2C—serotonin 2c (5-HT2c) receptor, HTR3A—serotonin 3a (5-HT3a) receptor, HTR5A—serotonin 5a (5-HT5a) receptor, HTR6—serotonin 6 (5-HT6) receptor, HTR7—serotonin 7 (5-HT7) receptor, F2—thrombin, F10—thrombin and coagulation factor X, PLAU—urokinase-type plasminogen activator.

## Data Availability

Any unpublished raw data associated with this research are available by contacting the corresponding author J.P.
